# Effects and Mechanism of Acupuncture Based on the Principle of Meridians

**DOI:** 10.1155/2013/684027

**Published:** 2013-11-27

**Authors:** Wei-Bo Zhang, Andrew Wu, Gerhard Litscher, Younbyoung Chae

**Affiliations:** ^1^China Academy of Chinese Medical Sciences, Beijing 100700, China; ^2^Five Branches University, San Jose, CA 95128, USA; ^3^Stronach Research Unit for Complementary and Integrative Laser Medicine, Research Unit of Biomedical Engineering in Anesthesia and Intensive Care Medicine, TCM Research Center, Medical University of Graz, 8036 Graz, Austria; ^4^Acupuncture and Meridian Science Research Center, College of Korean Medicine, Kyung Hee University, Seoul, Republic of Korea

This special issue concerns acupuncture effects related with meridians. Out of the 34 submitted papers, 15 papers were accepted to be published (acceptance rate of 44.1%). Of the 15 papers, five papers focused on the clinical effects related to meridians. In a study by J.-H. Jeong et al., a simple acupoint flowchart based after meridian theory was used to direct the acupuncture treatment on 102 dogs with various diseases, and good curative effects were achieved in their study that was entitled “*Simple acupoints prescription flowchart based on meridian theory: a retrospective study in 102 dogs.*” Two papers reported on meridian-sinew therapy for knee osteoarthritis. S. Wei et al. observed better results in 90 cases using meridian-sinew release, as compared to normal acupuncture and drug treatments, in their study that was entitled “*Evaluating meridian-sinew release therapy for the treatment of knee osteoarthritis.*” In an RCT study, Y.-S. Liu et al. compared 97 patients treated with long-round needle therapy to 95 control patients treated with drugs and found a better effect from long-round needle treatment both immediately after the treatment and at the 3-month followup. The effect mechanism was analyzed according to the relationship between the meridian sinew and the fluid meridian channels in their study that was entitled “*Observation on pain release by long-round needle therapy in knee osteoarthritis related with meridian-sinews theory.*” S.-H. Lee et al. performed a network analysis of the acupuncture points used in treating low back pain in which modified mutual information value was calculated to illustrate the technique of acupoint combination in acupuncture treatment in their study that was entitled “*Network analysis of acupuncture points used in the treatment of low back pain.*” I.-S. Lee et al. summarized 37 clinical studies on the treatment of low back pain and found that the most frequently used acupoints were BL23 (51%), BL25 (43%), BL24 (32%), BL40 (32%), BL60 (32%), GB30 (32%), BL26 (28%), BL32 (28%), and GB34 (21%). A visualized meridian chart was drawn based on the data to understand the origin of meridians in their study that was entitled “*Visualization of the meridian system based on biomedical information about acupuncture treatment*”. All of five papers confirmed the importance of meridian theory in clinical acupuncture.

The second group of papers focused on various effects of acupuncture when stimulating acupoints and meridians. W.-B. Zhang et al. found an increase of blood perfusion when needling a point on a meridian but not on an acupoint, as compared to needling a point off meridian but at an acupoint level, which proved the ancient theory that missing a point is less important than missing a meridian in their study that was entitled “*Comparison of acupuncture effect on blood perfusion between needling nonacupoint on meridian and needling nonacupoint off meridian.*” Gerhard Litscher et al. found that, when acupressing Xiyangguan (GB33), the regional oxygen saturation at the deeper knee tissue increased significantly while no considerable change occurred in the opposite control area, implying a meridian-related effect in their study that was entitled “*Acupressure at the meridian acupoint xiyangguan (GB33) influences near-infrared spectroscopic parameters (regional oxygen saturation) in deeper tissue of the knee in healthy volunteers.*” 

Four papers focused on the mechanism of acupuncture. W.-B. Zhang et al. studied the mechanism of acupuncture in channel dredging. A channel blockage model was made by injecting gel into a low hydraulic resistance channel (LHRC) in minipigs resulting in hyperalgesia. Acupuncture was applied to reduce the hydraulic resistance in the LHRC, which verified the ancient meridian theory that acupuncture can dredge a channel to relieve pain in their study that was entitled “*Induction of hyperalgesia in pigs through blocking low hydraulic resistance channels and reduction of the resistance through acupuncture: a mechanism of action of acupuncture.*” Z.-D. Cheng et al. observed the impact of acupuncture along meridians on the protein expression of chloride channels in myocardial ischemia rats in their study that was entitled “*The impacts of along-channel acupuncture on the protein expressions of the chloride channel of the rats with myocardial ischemia.*” J. Lu et al. reported that acupuncturing Baihui (Du20) and Neiguan (PC6) can increase the ratio of p-ERK1/2 to ERK1/2 and the ratio of p-CREB to CREB at the hippocampus and prefrontal cortex in rats with chronic unpredictable mild stress in their study that was entitled “*Acupuncture activates ERK-CREB pathway in rats exposed to chronic unpredictable mild stress.*” Both studies imply that acupuncture may also influence protein expression. M.-F. Luo et al. observed that recruitment and migration of mast cells along blood vessels and nerves to form a compound structure at acupoint sites after electroacupuncture (EA) were applied, implying that a dynamic compound structure may play an important role in the effect of acupuncture in their study that was entitled “*Study on the dynamic compound structure composed of mast cells, blood vessels, and nerves in rat acupoint.*”

The relationship between meridians and internal organs was addressed in four papers. W.-T. Zhou et al. developed a channel stasis model in minipigs by injecting gel into LHRC of the stomach meridian and found a distension of stomach and intestine after one or two months while no change was observed in control pigs that were injected with saline in their study that was entitled “*Pathological changes in Internal organs after blocking low hydraulic resistance channels along the stomach meridian in pigs.*” S. Hu et al. developed an intestinal barrier injury by ischemic reperfusion. EA was applied at ST36 and nonmeridian points in various conditions. EA at ST36 attenuated the systemic inflammatory response and remote organ injury by protection of the intestinal barrier under the condition of an intact vagus nerve and cholinergic system significantly better than acupuncturing at nonmeridian points in their study that was entitled “*Electroacupuncture at zusanli (ST36) prevents intestinal barrier and remote organ dysfunction following gut ischemia through activating the cholinergic anti-inflammatory-dependent mechanism.*” X. Shi et al. also studied the impact of acupuncture at ST36 and nonmeridian points on rats after a fatal hemorrhagic shock. They found that EA at ST36 significantly improved the blood pressure and increased the early survival rate, as compared to EA at nonmeridian points. EA can impact internal organs at specific meridian/acupoint sites in their study that was entitled “*The influence of zusanli and nonmeridian acupuncture points on the survival rate and intestinal tissue features after fatal hemorrhagic shock in rats.*” K.-M. Shin et al. studied the effect of acupuncturing Siguan (bilateral LI4 and LR3) on gastrointestinal motility in healthy people after administering mosapride citrate. They found that acupuncturing Siguan can significantly reduce increased gastrointestinal motility, as compared to sham acupuncture in their study that was entitled “*Effect of siguan acupuncture on gastrointestinal motility: a randomized, sham-controlled, crossover trial.*” 

 In this special issue, we have provided an overview of the majority of studies related to the effects and mechanisms of acupuncture based on principal meridians.

*Wei-Bo Zhang* *Wei-Bo Zhang* *Andrew Wu* *Andrew Wu* *Gerhard Litscher* *Gerhard Litscher* *Younbyoung Chae* *Younbyoung Chae*

